# Effect of dog-owner interaction on post-operative pain perception and stress of dogs and variability in their behavioural patterns

**DOI:** 10.1017/awf.2024.49

**Published:** 2024-11-04

**Authors:** Deborah Lazard, Déborah Temple, Edgar Palma, Aurora C. Diaz, Maria B. Rosado, Mariana Medrano, Roberto Ruiz, Marta Amat

**Affiliations:** 1 Faculty of Veterinary Medicine, Universitat Autònoma de Barcelona, UAB, Spain; 2 Veterinary Clinic of Dr Palma, Mexico City, Mexico; 3 Condesa Pet Center, Mexico City, Mexico; 4 Canine Psychology, Mexico City, Mexico; 5 AWEC Animal Welfare Education Center, Universitat Autònoma de Barcelona, UAB, Spain

**Keywords:** animal welfare, canine behavioural patterns, dog-owner interaction, ovariohysterectomy, pain management, post-operative pain

## Abstract

Pain, a multifaceted condition associated with actual or potential tissue damage, transcends nociception and is characterised as a subjective, sensory, and emotional experience. Extensive literature describing the adverse effects of untreated post-surgical pain emphasises the necessity of a comprehensive pain management protocol, incorporating both pharmacological and non-pharmacological strategies to ensure successful patient outcomes. The present study aimed to determine whether a positive dog-owner interaction influences post-operative pain perception and stress (POPPS), as well as behavioural inactive rate variability in bitches that underwent elective surgery. Randomly selected bitches (n = 18) underwent ovariohysterectomy. Eight bitches experienced a 45-min visit post-surgery (VPS) characterised by positive dog-owner interaction, while the remaining ten did not (NVPS). Utilising the validated Short Form of the Glasgow Composite Measure Pain Scale (CMPS-SF) to assess acute pain in dogs via stress-related behaviours, a significant decrease in POPPS was evident in the VPS group after the 45-min dog-owner interaction at T3 (1 h after post-sedation recovery), in contrast to the NVPS group. CMPS-SF-associated descriptive items ‘Nervous/Anxious/Fearful’ and ‘Happy Content or Happy and Bouncy’ decreased and increased, respectively, with dog-owner positive interaction in the VPS group. The inactivity rate was significantly lower in VPS bitches after the post-surgery 45-min dog-owner interaction than in NVPS bitches. This preliminary study suggests that the owner’s presence reduces POPPS and may improve the dogs’ welfare while undergoing routine surgeries.

## Introduction

Effective pain management in veterinary practice is crucial for successful outcomes, as highlighted in the 2022 *AAHA/AAFP Pain Management Guidelines for Dogs and Cats* (Gruen *et al.* 2022). Addressing acute post-surgical pain is vital for a patient’s recovery, and failure can lead to consequences such as sensitisation, neuropathic pain and maladaptive pain (Poleshuck *et al.* 2006; Nikolajsen & Minella 2009; Johansen *et al.* 2012; Masselin-Dubois *et al.* 2013). Inadequate pain control may also result in delayed healing, reduced food intake, sleep disturbances, compromised mobility, post-operative cognitive dysfunction (POCD), and changes in species-specific activities (Kitchell 1987; McGuire *et al.* 2006; Jirkof 2017; Nimmo *et al.* 2017).

Although spaying and neutering are known to cause moderate pain (Hardie *et al.* 1997; Siracusa *et al.* 2010; Slingsby *et al.* 2011; Srithunyarat *et al.* 2016), this pain is not always adequately prevented (Simon *et al.* 2017). Studies carried out on cats and dogs have shown significant variations in the frequency of analgesic use depending on species, sex, geographical location, and procedural stages (Farnworth *et al.* 2014; Lorena *et al.* 2014; Perret-Gentil *et al.* 2014; Simon *et al.* 2017). Factors such as insufficient assessment and recognition of pain (Hugonnard *et al.* 2004; Williams *et al.* 2005), drug-induced adverse effects, and a lack of clinical familiarity and experience in prescribing and administering opioids and NSAIDs continue to hinder their utilisation in veterinary practice.

In addition to the challenges in analgesic use, research suggests that effective pain management goes beyond pharmaceutical interventions alone, emphasising the significant influence on pain of cognitive and emotional factors (Epstein *et al.* 2015; Luna *et al.* 2015; Peters 2015). Human and animal studies consistently reveal a robust connection between psychological and emotional states and the experience of chronic or acute pain (Schlereth & Birklein 2008; Abdallah & Geha 2017; Zanini *et al.* 2018; Michaelides & Zis 2019; Kang *et al.* 2022; Sun *et al.* 2023). The intricate relationship between emotional states and pain is such that behavioural observation-based pain scales fail to distinguish between them (Siracusa *et al.* 2008), as they exert a similar influence on behaviour. Moreover, behavioural pain scales such as the GPS (Glasgow Pain Scale) and the MGPS (Modified Glasgow Pain Scale) are influenced by the psychological stress experienced by dogs during pre-surgery (Siracusa *et al.* 2008; de Santana *et al.* 2020). Therefore, values of behavioural pain scales are to be interpreted as representing psychological stress behaviours that include nociception components under specific circumstances.

Furthermore, perioperative emotional distress and short-term/sub-acute stress can significantly heighten pain levels and reduce tolerance, potentially leading to hyperalgesia and increased reliance on analgesia (Munafò & Stevenson 2001; Caumo *et al.* 2002; Ford *et al.* 2008; Jackson *et al.* 2016; Sun *et al.* 2023). In contrast, approaches that foster positive emotions and mitigate stress, fear, and anxiety, such as non-pharmacological interventions (including acupuncture, yoga, meditation, psychotherapy, and even the placebo effect), have demonstrated efficacy in reducing pain perception (Villemure & Bushnell 2002; Castiglioni *et al.* 2009; Bushnell *et al.* 2013; Nakata *et al.* 2014; Zanini *et al.* 2018). While no existing reports have specifically investigated the owner’s potential role in mitigating dogs’ pain perception, a wealth of literature underscores the significant impact owners have on their dogs’ emotional regulation (Hare & Tomasello 2005; Kaminski *et al.* 2012; Prato Previde & Valsecchi 2014).

Additionally, research indicates that owners’ presence and interaction during veterinary exams, known to be particularly stressful for dogs (Edwards *et al.* 2019), lead to a reduction in psychological and physiological stress indicators in the dogs (Csoltova *et al.* 2017; Stellato *et al.* 2020; Girault *et al.* 2022; Helsly *et al.* 2022). Moreover, a recent study by Camarasa *et al.* (2023) demonstrated that dogs with brachycephalic obstructive airway syndrome, discharged with owners on the same day as surgery, experienced fewer post-operative complications than those kept overnight.

This study sought to investigate the impact of dog-owner interaction on dogs’ pain perception and stress (PPS). The hypothesis posits that implementing such a protocol during ovariohysterectomy (OVH) in bitches mitigates post-operative pain perception and stress (POPPS). Additionally, we evaluated the interaction’s influence on the inactivity rate behaviours which were recognised as indicators of post-operative psychogenic stress in dogs (Siracusa *et al.* 2008).

## Materials and methods

### Ethical statement

Our research required neither licences nor permission from ethical review bodies in Spain or Mexico for the following reasons: (a) all bitches in this study (n = 18) were selected exclusively because they had an elective ovarian hysterectomy scheduled at one of the two private clinics where this study was conducted; (b) informed consent was obtained from all owners; (c) direct intervention was performed exclusively by the veterinarian surgeons in both clinics before, during or in the recovery of the surgical procedure; (d) all decisions concerning the well-being and welfare of the subjects were made exclusively by these veterinarians; and (e) each clinic used a different anaesthesia and analgesia protocol, as well as rescue analgesia, based on their individual standard operating procedures. Neither of the two clinics had used specific pain scales prior to this study.

### Study design

Bitches were assigned to one of two groups (VPS vs NVPS) based on their owners’ decision upon arrival at the clinic, i.e. to have a 45-min post-surgery positive interaction with their dogs or not, respectively. All subjects were assessed for PPS using the Validated Short Form of the Glasgow Composite Measure Pain Scale (CMPS-SF; Reid *et al.* 2007) and recorded for 16 min at three-time-points: (T1) upon arrival at the clinic after being placed in crates in the intensive care unit (ICU) without owners’ presence (OVH was performed between 1 and 2 h after arrival); (T2) post-sedation recovery, between 2 h 30 min and 3 h 30 min post-surgery; and (T3) 1 h after T2. Positive interaction lasted 45 min for all post-surgery bitches from the VPS group. This interaction involved bitches and owners engaging in an isolated environment with minimal movement of people or other dogs and low noise levels. Owners were instructed: (a) to refrain from interfering with their dog’s natural behaviours and movements and avoid encouraging specific actions or movements in them; and (b) to express vocal affection and provide gentle caresses whenever they felt inclined or when their dog requested it.

### Study animals

Eighteen randomly selected adult bitches (mean age: 36 [± 7.9] months, mean weight: 14 [± 2.4] kg) underwent elective OVH in two private clinics in Mexico City (Table 1). The group included purebred and mixed-breed dogs, all of whom had lived with human families for at least three months prior to the study. All the dogs were in good health, as confirmed by their respective veterinarians. Among the total group of 18 bitches, eight were visited post-surgery (VPS) by their owners (six underwent elective surgery at clinic A and two at clinic B), and ten were non-visited post-surgery (NVPS) by their owners (five underwent elective surgery at clinic A and five at clinic B) (Table 1).

**Table 1.** Characteristics of study animals in terms of age, breed, weight, clinic and whether they were visited (VPS) or non-visited (NVPS) post-surgery by their owners

SUBJECT	(VPS)/ (NVPS)*	AGE(MONTHS)	BREED	WEIGHT (KG)	CLINIC
1	VPS	8	MIXED	5	A
2	VPS	120	YORKIE	2.6	A
3	VPS	12	MIXED	17.3	A
4	VPS	8	PUG	5.3	A
5	VPS	36	LABRADOR	30	A
6	VPS	12	MIXED	15	A
7	VPS	9	CHIHUAHUA	3.7	B
8	VPS	96	MIXED	17.7	B
9	NVPS	36	BORDER COLLIE	21	A
10	NVPS	48	AUSTRALIAN CATTLE DOG	27	A
11	NVPS	72	LABRADOR	27.1	A
12	NVPS	36	DACHSHUND	4.2	A
13	NVPS	36	BOXER	20	A
14	NVPS	12	DACHSHUND	6.9	B
15	NVPS	5	MIXED	3.5	B
16	NVPS	72	FRENCH BULLDOG	10.4	B
17	NVPS	18	AMERICAN STAFFORDSHIRE TERRIER	30.6	B
18	NVPS	17	MIXED	6.2	B

* VPS: Visited post-surgery; NVPS Non-visited post-surgery

Both clinics followed specific analgesia and anaesthesia protocols: clinic A (11/18 bitches) used Xylazine HCl (Virbac México SA de CV, Mexico) (1 mg kg^–1^) for pre-anaesthesia, Propofol (Aculife Healthcare PVT Ltd, India) (1 mg kg^–1^) for anaesthesia induction, and Isoflurane (Piramal Healthcare Ltd, India) as an inhaled anaesthetic. Analgesia was provided with Tramadol HCL (Pisa Agropecuaria SA de CV, México) (3 mg kg^–1^) at the end of surgery and subsequently every 8 h. In contrast, clinic B (7/18 bitches) administered Tiletamine HCL and Zolazepam HCL (1 mg kg^–1^) and Dexmedetomidine (0.5 mg kg^–1^) for pre-anaesthesia, Propofol (4 mg kg^–1^) for anaesthesia induction, and Isoflurane as an inhaled anaesthetic. Analgesia included Buprenorphine (0.15 mg kg^–1^) and Meloxicam (0.1 mg kg^–1^) at the end of surgery and every 8 h.

### PPS Assessment

Pain Perception and Stress (PPS) were evaluated using the CMPS-SF, a validated tool for assessing acute pain in dogs (Reid *et al.* 2007). Despite its primary focus on pain assessment, the CMPS-SF also incorporates stress-related behaviours that, in certain contexts, may indicate pain (Hellyer & Gaynor 1998; de Santana *et al.* 2020). In this manuscript, both pain perception (PPS) and post-operative pain perception (POPPS) encompass both pain and psychological stress.

The CMPS-SF includes six behavioural categories: vocalisation (four items); attention to the wound (five items); mobility (five items); response to touch (six items); demeanour (five items); and posture/activity (five items). Descriptive items within each category were scored on a binary system (1 for present, 0 for absent). The summed scores (with a maximum of 24 points) had a recommendation for rescue analgesia when 6 points or more were accrued (Reid *et al.* 2007).

The assessment of PPS was conducted by an independent behaviourist who had no direct contact with the subjects. Specifically, the evaluation of the CMPS-SF category ‘attention to wound’ was performed by the local veterinary surgeon at each clinic. Observations made at the clinic were not blinded and subsequently rechecked via video analysis.

Bitches requiring rescue analgesic treatment, based upon clinicians’ decisions, were excluded from the study. Therefore, the sample size (n = 18) represents bitches that did not require rescue analgesia.

### Inactive rate

Bitches were each recorded for 16 min during T1, T2, and T3 using an iPhone 12 (Apple, Inc), with scan sampling conducted every 2 min. Behaviours were classified as either active or inactive (Table 2) in nine instantaneous scan samples at each of the three time-points, using a binary scoring system (0 for active, 1 for inactive). These observations allowed for the calculation of an inactivity rate, expressed as the percentage of the nine visual samples for each time point.

**Table 2.** Active and inactive patterns of study animals as per Siracusa *et al.* (2008)

Active	Standing: Positioned on four paws in contact with the ground, two on the ground, and two against the inner wall of the cage.
	Sitting: Front leg pads are on the floor, with the front legs straight and the rump directly on the floor.
Inactive	Lying ventral: Positioned on the side with the body, but not the head, in complete contact with the ground, or with the ventral side and legs in contact with the ground.
	Lying sideways: Positioned fully on its side, with one side of the dog in complete contact with the ground.

### Statistical analysis

Data were analysed using the SAS® software (SAS® Institute Inc, Cary, NC, USA). CMPS-SF score and inactive rate were analysed using a generalised mixed model for repeated measures. A Poisson distribution for count data was applied for CMPS-SF score. A binomial distribution was applied for the inactivity rate. The model included the fixed effect of the clinic (A and B), the treatment group (VPS and NVPS), the time-point (T1, T2 and T3), and the interaction between treatment group and time-point. The dog was introduced as a repeated measure in the random statement. The least square means for these effects were estimated to provide adjusted comparisons across the groups and time-points. Pair-wise comparisons were conducted, and *P*-values adjusted using Tukey corrections to control for multiple testing. The significance level was set at *P* < 0.05.

## Results

### Pain Perception and Stress (PPS) assessed through the CMPS-SF

**Figure 1.**
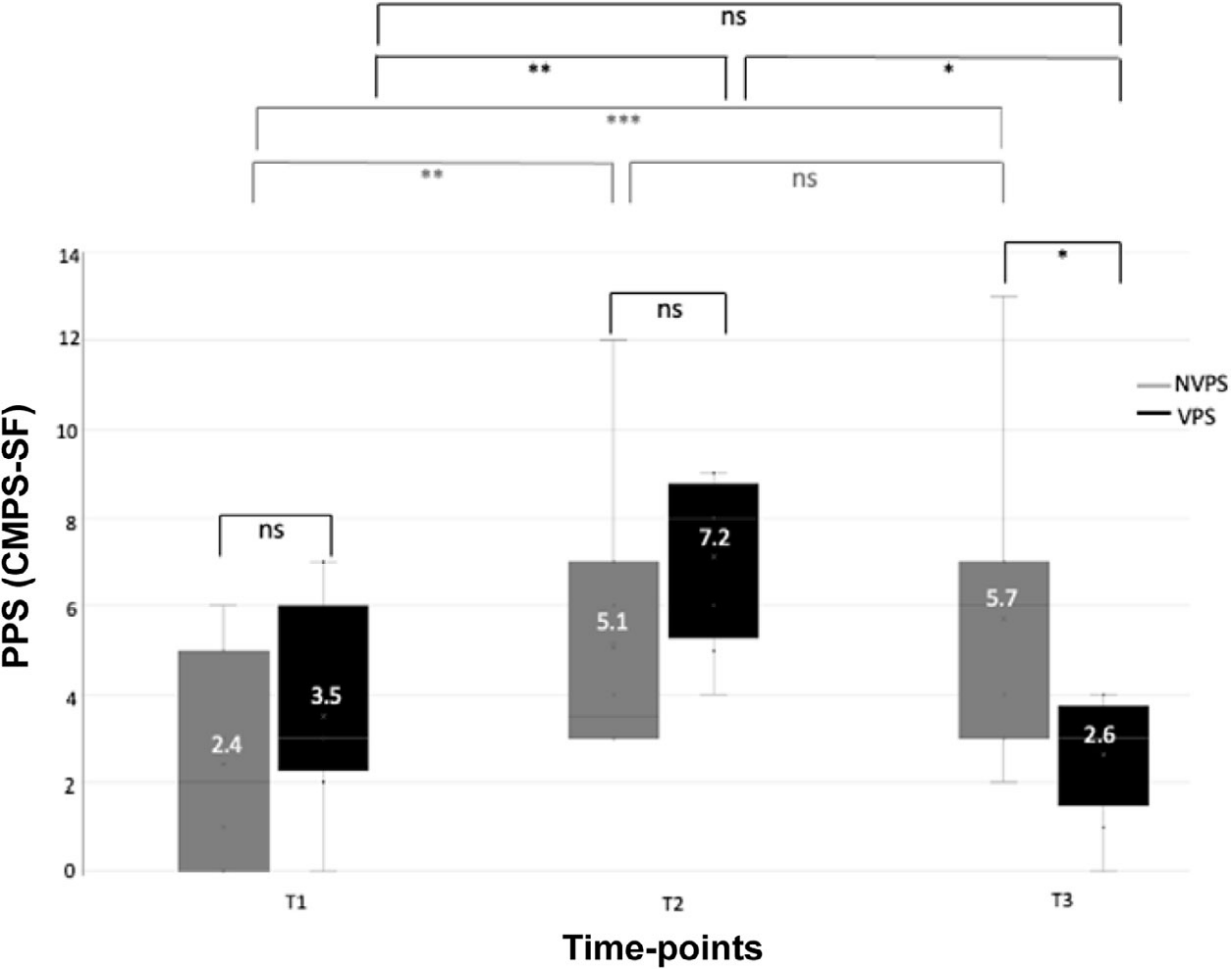
Boxplots showing the comparisons of PPS measured by CMPS-SF values between groups (NVPS: light grey, n = 10; VPS: dark grey, n = 8) at three different time-points: T1 (arrival at the clinic); T2 (post-sedation recovery); and T3 (60 min after T2 with or without dog-owner interaction). The number above each X in the box represents the mean of PPS (CMPS-SF). PPS: pain perception and stress; CMPS-SF: short-form Glasgow Composite Measure Pain Scale; NVPS: non-visited post-surgery; VPS: visited post-surgery; NS: non-significant; * *P* < 0.05; ** *P* < 0.01; *** *P* < 0.001.

A significant interaction between the treatment group and the time-point for the CMPS-SF (*P* < 0.001) was detected (Figure 1). Upon arrival at the clinic (T1), the VPS and NVPS groups exhibited no significant differences in basal CMPS-SF scores (*P* > 0.05) (Figure 1). The OVH procedure increased the CMPS-SF scores in both groups of bitches by more than two-fold, with no significant differences between treatment groups at T2 (between 2 h 30 min and 3 h 30 min post-surgery) (Figure 1). However, the VPS group presented significantly lower CMPS-SF scores than the NVPS group at T3 (60 min after T2) following dog-owner interaction (*P* = 0.013; Figure 1).

Differences between periods for the VPS and NVPS groups exhibited a significant increase for both groups in CMPS-SF values at T2 as a consequence of surgery (*P* = 0.007 for the NVPS group and *P* = 0.001 for the VPS group) (Figure 1). While between T2 and T3, the NVPS group remained with stable CMPS-SF values; in the same time-frame after a 45-min dog-owner positive interaction, the VPS group exhibited a significant decrease (*P* < 0.001) (Figure 1), returning to CMPS-SF basal values.

Bitches underwent surgery in two different clinics, A and B. The clinic (A or B) did not significantly affect the CMPS-SF, irrespective of variations in anaesthetic/analgesic protocols. Similar patterns of PPS were detected at clinics A and B, consistent with those observed with the entire VPS and NVPS groups.

At the basal level (T1), VPS and NVPS groups in both clinics included bitches that exhibited CMPS-SF basal values around the rescue threshold (≥ 6/24) (data not shown). The OVH procedure yielded high CMPS-SF scores in the VPS and NVPS bitches in both clinics (in clinic A: 7.5 [± 0.67] in the VPS group and 5.6 [± 1.78] for the NVPS group, and in clinic B: 6.0 [± 2.0] in the VPS group and 4.6 [± 0.81] in the NVPS group). At T3 (60 min after T2), following dog-owner interaction, the VPS group presented lower CMPS-SF scores compared to the NVPS group at both clinics (at clinic A: 3.0 [± 0.45] for VPS bitches vs 6.6 [± 1.75] for the NVPS bitches and in clinic B: 1.5 [± 1.5] for VPS bitches vs 4.8 [± 0.97] for the NVPS bitches).

Differences between periods for the VPS and NVPS groups in both clinics were consistent with those observed for the entire population. Differences in anaesthetic/analgesic protocols rendered a greater POPPS increase in clinic A compared to clinic B in the VPS and NVPS groups of bitches (in clinic A: from 3.0 [± 9.3] at T1 to 7.5 [± 0.67] at T2 in the VPS group and from 2 [± 0.14] to 5.6 [± 1.78] for the NVPS group, and in clinic B: from 5.0 [± 2.0] to 6.0 [± 2.0] in the VPS group and from 2.8 [± 1.02] to 4.6 [± 0.81] in the NVPS group); however, VPS bitches of both clinics A and B showed a decrease in CMPS-SF values after dog-owner interaction at T3 of at least 4 points (at clinic A: from 7.5 [± 0.67] at T2 to 3.0 [± 0.45] at T3 and in clinic B: from 6.0 [± 2.0] to 1.5 [± 1.5] at T3). At both clinics, NVPS bitches depicted a small increase in POPPS from T2 to T3.

### Individual PPS Assessment

**Figure 2.**
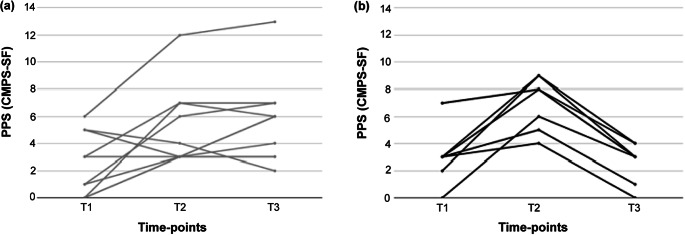
PPS measured with CMPS-SF for each individual bitch at three time-points: T1 (arrival at the clinic); T2 (post-sedation recovery); and T3 (60 min after T2). The measurements are presented based on the treatment group: NVPS (a) representing no dog-owner interaction, and VPS (b) representing dog-owner interaction between T2 and T3. PPS: pain perception and stress; CMPS-SF: short-form Glasgow Composite Measure Pain Scale.

Figure 2 shows changes in PPS assessed by the CMPS-SF for each individual bitch across time-points, relative to the treatment group, NVPS, or VPS (Figure 2 left and right, respectively). Basal CMPS-SF values varied largely in both groups from scores that ranged from 0 to 7 points. Five out of 18 (27.7%) of the total group of bitches exhibited basal CMPS-SF scores between 5 and 7 points, meeting or surpassing the suggested rescue threshold of ≥ 6/24, indicating different levels of perioperative stress. OVH yielded CMPS-SF values in the NVPS group ranging from 3 to 12 points, with 40% (4/10) of them near or surpassing the rescue threshold value. In the VPS group, values ranged from 4 to 9 points, with 87.5% (7/8) of them near or surpassing the rescue threshold value (Figure 2, left and right, respectively). After 1 h at T3, NVPS bitches’ CMPS-SF values range had expanded from 2 to 12 points, with 60% (6/10) of the group with CMPS-SF meeting or surpassing the rescue threshold value. In contrast, at this time, in the VPS group, the CMPS-SF values concentrated between 0 and 4 points (Figure 2, left and right side, respectively).

### CMPS-SF-associated descriptive items

The descriptive items associated with CMPS-SF are presented as percentages of bitches exhibiting each item in the VPS and NVPS groups at the T1, T2, and T3 time-points (Table 3). Due to the lack of variability in the data, no statistical analysis was conducted. At the pre-surgery time point (T1), 12.5% of dogs in the VPS group were classified as ‘Comfortable’, compared to 70% in the NVPS group. At the post-surgery time-point (T2),12.5% of the VPS bitches were classified as ‘Quiet’, compared to 80% of the NVPS bitches. By T3, 87.5% of the VPS bitches displayed behaviours described as ‘Happy and Content or Happy and Bouncy’, while none of the NVPS bitches exhibited these behaviours. Conversely, at T3, 12.5% of the VPS bitches were classified as ‘Quiet’, compared to 90% of the NVPS bitches (Table 3).

**Table 3.** Comparison between time-points of the percentage of VPS and NVPS bitches displaying CMPS-SF-associated descriptive items

	% Pre-surgery Bitches	% Post Surgery Bitches	% Bitches after 45 min dog-owner interaction	% Pre-surgery Bitches	% Post Surgery Bitches	% Bitches without dog-owner interaction
IS THE DOG?	(VPS) T1**	(VPS) T2**	(VPS) T3**	(NVPS) T1**	(NVPS) T2**	(NVPS) T3**
*A 1: Vocalisation						
Quiet	50%	62.50%	75%	80%	70%	70%
Crying	25%	12.50%	25%	20%	20%	30%
Groaning	25%	12.50%	0%	0%	0%	0%
Screaming	0%	12.50%	0%	0%	10%	0%
*A 2: Attention to wound						
Ignoring wound	100%	87.50%	100%	100%	90%	90%
Looking wound	0%	0%	0%	0%	10%	0%
Licking wound	0%	12.50%	0%	0%	0%	10%
*B 3: Mobility						
Normal Mobility	100%	87.50%	100%	100%	90%	90%
Lame Mobility	0%	12.50%	0%	0%	0%	0%
It refuses to move	0%	0%	0%	0%	10%	10%
*C 4: Response to touch						
Doing Nothing	100%	50%	62.50%	100%	60%	60%
* Looking Around	0%	25%	12.50%	0%	0%	0%
Flinch	0%	0%	12.50%	0%	0%	0%
Growling	0%	25%	12.50%	0%	40%	30%
Crying	0%	0%	0%	0%	0%	10%
*D 5: Demeanour						
Happy Content or Happy Bouncy	25%	25%	87.50%	20%	0%	0%
Quiet	12.50%	12.50%	12.50%	50%	80%	90%
Nervous/Anxious/Fearful	62.50%	62.50%	0%	30%	20%	10%
*D 6: Posture/Activity						
Comfortable	12.50%	12.50%	37.50%	70%	10%	0%
Unsettled	12.50%	0%	12.50%	10%	20%	20%
Restless	75%	25%	37.50%	20%	40%	40%
Hunched or Tense	0%	62.50%	12.50%	0%	30%	40%

* CMPS-SF Behavioural categories

** Time-points: (T1) arriving at the clinic; (T2) after recovering from sedation; and (T3) 60 min after T2 with (VPS) or without (NVPS) post-surgery dog-owner interaction

Surgery affected the percentage of bitches in the VPS group exhibiting the CMPS-SF-associated descriptive item ‘Hunched or Tense’, increasing from 0% at T1 to 62.5% at T2. In the NVPS group, the percentage of bitches classified as ‘Comfortable’ decreased from 70% at T1 to 10% at T2 (Table 3). Following a 45-min dog-owner interaction (T3 vs T2), changes were observed in the VPS group, with the percentage of bitches classified as ‘Happy and Content or Happy and Bouncy’ increasing from 25% at T2 to 87.5% at T3, and the percentage of those classified as ‘Nervous/anxious/fearful’ decreasing from 62.5% at T2 to 0% at T3 (Table 3). No significant changes were observed in the NVPS group between T2 and T3 (Table 3).

Additionally, differences in the percentage of bitches displaying CMPS-SF-associated descriptive items between T1 and T3 were noted. Specifically, in the VPS group, the percentage of bitches classified as ‘Happy and Content or Happy and Bouncy’ increased from 25% at T1 to 87.5% at T3, while those classified as ‘Nervous/anxious/fearful’ decreased from 62.5% at T1 to 0% at T3 (Table 3). In the NVPS group, the percentage of bitches classified as ‘Comfortable’ decreased from 70% at T1 to 0% at T3.

### Variability in the inactivity rate

**Figure 3.**
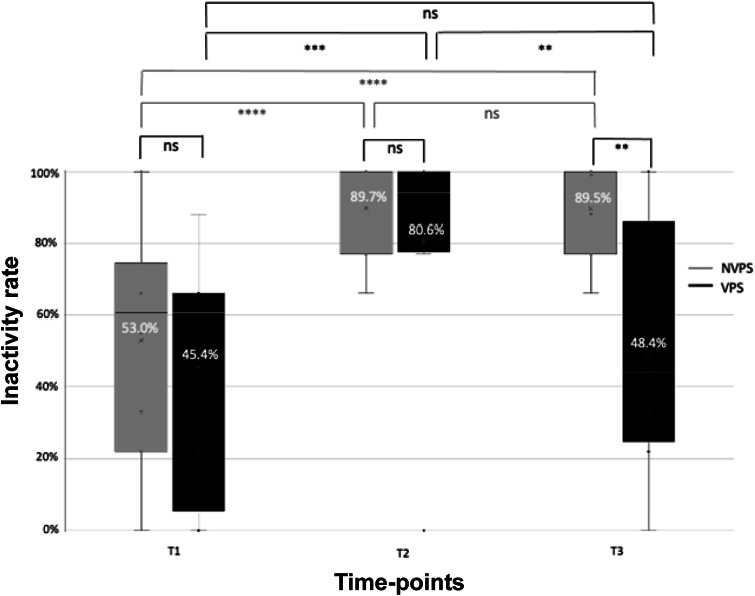
Boxplots showing the comparisons of inactivity rate between groups (NVPS: light grey, n = 10; VPS: dark grey, n = 8) at three different time-points: T1 (arrival at the clinic); T2 (post-sedation recovery); and T3 (60 min after T2 with or without dog-owner interaction). Inactive rate is expressed as a percentage of times the dogs were observed lying in a ventral or sideways posture (9 samples at 2-min intervals per time-point). The numbers over the X in each box represent the mean value of inactivity rate for each group. NVPS: non-visited post-surgery; VPS: visited post-surgery; NS: non-significant; * *P* < 0.05; ** *P* < 0.01; *** *P* < 0.001.

A significant interaction was detected between the treatment group and the time-point for the inactivity rate (*P* = 0.0117; Figure 3). Both treatment groups presented similar inactivity rates at T1 (upon arrival at the clinic) and T2 (between 2 h 20 min and 3 h 30 min post-sedation). However, at T3 (1 h after T2), VPS presented a significantly lower inactivity rate than NVPS (*P* = 0.0046) (Figure 3).

A significant increase (*P* < 0.001) in the inactivity rate was observed in both groups between pre- and post-surgery (T2 vs T1) (Figure 3). The NVPS group maintained a high inactivity rate between T2 and T3 (1 h after T2) with no significant differences between treatment points (*P* > 0.05). After a 45-min dog-owner interaction between T2 and T3, the inactivity rate for the VPS group decreased significantly (*P* = 0.005), returning to its basal (T1) level (Figure 3).

No significant effect on inactivity rate was detected between bitches that underwent surgery in clinic A or B, irrespective of variations in anaesthetic/analgesic protocols. Similar patterns of inactivity rate were detected at clinics A and B, consistent with those observed with the entire VPS and NVPS groups.

### Inactivity rate for individual bitches

**Figure 4.**
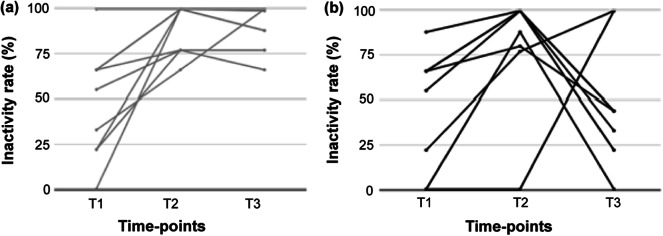
Inactivity rate measured for each individual bitch at three time-points: T1 (arrival at the clinic); T2 (post-sedation recovery); and T3 (60 min after T2). The measurements are presented based on the treatment group: NVPS (a) representing no dog-owner interaction, and VPS (b) representing dog-owner interaction between T2 and T3.

Figure 4 shows the changes in the inactivity rate for each dog across time-points and depending on the treatment group. At T3, following a 45-min interaction with owners, 75% (6/8) of the VPS group exhibited a decrease in the inactive rate, while the remaining 25% (2/8) showed an increase. Such individual variability in inactivity rate was not observed for NVPS bitches since all maintained a virtually identical inactivity rate in T3 as in T2 (Figure 4).

## Discussion

This study aimed to examine the influence of positive interactions between bitches and their owners on post-operative pain perception and stress (POPPS), as well as inactivity rate. Pain perception and stress (PPS) were evaluated using scores from the Short Form of the Glasgow Composite Measure Pain Scale (CMPS-SF), a validated behavioural assessment tool for assessing acute pain in dogs (Reid *et al.* 2007). The CMPS-SF considers stress-related behaviours that may indicate pain in specific contexts (Hellyer & Gaynor 1998; de Santana *et al.* 2020), thus demonstrating high sensitivity to psychological stress as well.

The findings presented here strongly indicate that positive interactions between bitches that have undergone OVH surgery and their owners, following anaesthesia recovery, have a statistically significant effect on reducing POPPS and decreasing inactivity patterns.

As previously discussed by Luna *et al.* (2015) and de Santana *et al.* (2020), dogs’ experiences of stress have been demonstrated to significantly impact CMPS-SF scores. Our results agree; here, upon arrival at the clinic, 27.8% (5/18) of the total dog group displayed CMPS-SF scores ranging from 5/24 to 7/24 points. It is important to note that while these scores fall near the CMPS-SF rescue threshold of ≥ 6/24, they do not indicate perceived pain, as all dogs were healthy and had not undergone surgery or received medication. During this time, the CMPS-SF scores of the entire dog group were influenced by item descriptors such as ‘Crying’, ‘Growling’, ‘Quiet’, ‘Nervous, Anxious, or Fearful’, and ‘Restless’, all of which encompass emotional components. The OVH procedure significantly increased the VPS and NVPS CMPS-SF scores (2 h 30 min–3 h 30 min after surgery). No significant differences were observed between both groups in POPPS and in the inactivity rate, indicating that both responded to surgery without relevant differences. However, a positive 45-min post-surgery (T3 vs T2) interaction with their owners consistently resulted in a significant decrease in POPPS. This result was observed identically in both clinics. Each clinic deployed different analgesia and anaesthesia protocols. Despite subjects being treated with Tramadol hydrochloride exhibiting a more pronounced post-operative increase in CMPS-SF score compared to those receiving a multimodal approach with Buprenorphine and Meloxicam (Hellyer *et al.* 2007; Dongaonkar *et al.* 2019), we found that VPS bitches from both clinics experienced a statistically significant POPPS decrease compared to the NVPS group. The POPPS decrease, between T2 and T3, in the VPS group was characterised by changes in the percentage of bitches displaying the CMPS-SF-associated descriptive items ‘Happy and Content or Happy and Bouncy’ and ‘Nervous/Anxious/Fearful’.

POPPS in the VPS bitches decreased to the extent that the group exhibited a lower CPMS-SF value at the end of the study compared to the one for the same group upon arrival at the clinic. There were also changes in the percentage of bitches displaying the CMPS-SF-associated descriptive items ‘Happy and Content or Happy and Bouncy’ and ‘Nervous/Anxious/Fearful’, both of which have relevant emotional components and been described as part of the perioperative stress response in dogs subjected to elective surgery (Siracusa *et al.* 2008).

In the context of dogs, owners may play a role as distractors, mitigating stress and addressing emotional and cognitive components, thereby alleviating POPPS, which aligns with previous reports on non-pharmacological pain modulation interventions in humans, such as yoga or meditation, with promising results (Sharar *et al.* 2008; Nakata *et al.* 2014; Peters 2015; Li *et al.* 2019). As previously reported (Mariti *et al.* 2013; Petersson *et al.* 2017), our findings also highlight the crucial role owners play in promoting dogs’ well-being in stressful situations.

Parallels have been drawn between the bond that is shared by dogs and their owners and that of an infant and their caregiver, with shared behavioural and neuroendocrine characteristics (Hare & Tomasello 2005; Kaminski *et al.* 2012; Prato Previde & Valsecchi 2014; Nagasawa *et al.* 2015; Petersson *et al.* 2017). The reduction in POPPS observed in our study may echo findings in humans, where the presence and interaction of parents with their children during invasive procedures effectively reduced their pain scores (Filippa *et al.* 2021; Azar *et al.* 2022). We hypothesise that the observed reduction in POPPS among dogs that interacted with their owners may involve the neuropeptide, oxytocin, similar to infant-human interactions (Filippa *et al.* 2021).

Previous research has shown that the presence and affective interaction of dogs with owners can increase concentrations of oxytocin and other substances, including β-endorphin, prolactin, β-phenylethylamine, and dopamine in both dogs and humans (Odendaal & Meintjes 2003; Nagasawa *et al.* 2009; Handlin *et al.* 2012; Romero *et al.* 2014). Due to their evolutionary history (Miklósi 2009), it has been suggested that dogs and humans possess a unique ability to activate each other’s oxytocinergic systems, resulting in oxytocin-linked effects (Beetz *et al.* 2012). Even more so, animal models strongly support the idea that oxytocin has an analgesic effect, demonstrating increased pain tolerance and attenuation of acute pain (Rash *et al.* 2014). Additionally, the positive effects of oxytocin in alleviating manifestations of fear, stress, or anxiety can contribute significantly to minimising the perception of pain (Heinrichs *et al.* 2003; Poisbeau *et al.* 2018). More studies are needed to further explore this hypothesis.

Concerning the potential that any positive post-OVH human-dog interaction (not involving owners) could reduce POPPS, though not explored here (an avenue we consider valuable for future research), previous studies indicate that post-OVH-surgery bitches respond to handlers with a reduced tendency to move or actively interact with them (Hardie *et al.* 1997; Siracusa *et al.* 2008), which could mean that not all humans are the same from the dogs’ perspective and as has been demonstrated in previous studies (Mariti *et al.* 2013). However, differences between previous protocols and ours, such as the type of interaction and the small amount of time handlers spend with dogs, could be relevant and should be further explored.

Recognising that pain and stress can induce changes in animal behaviour, such as aggression, alterations in body posture, activity levels, and movement frequency (Hellyer *et al.* 2007; Camps *et al.* 2012; Lefman & Prittie 2019), our study aimed to investigate the influence of post-surgery dog-owner interaction on specific inactive behaviours, i.e. lying ventrally or sideways. Previous research has underscored the substantial impact of post-operative psychogenic stress on these behaviours in dogs undergoing elective surgery (Hardie *et al.* 1997; Siracusa *et al.* 2008).

We evaluated the inactivity rate between different time-points for each of the bitches in the study. Dog-owner interaction (T3 vs T2) yielded a significant difference in the inactivity rate in VPS bitches compared to NVPS. While the majority of the VPS bitches experienced a decrease in inactivity rate, a small proportion, which were in a rigid stance at T2, probably as a result of pain (Johnson 1991), exhibited an increase in inactivity rate after being reunited with their owner. Variability, as a consequence of dog-owner interaction, appeared as constant in the VPS group, in contrast to NVPS bitches where almost no variability in the inactivity rate between T2 and T3 was detected. Behavioural changes induced by dog-owner interaction during veterinary examination have been previously reported (Csoltova *et al.* 2017; Stellato *et al.* 2020; Girault *et al.* 2022; Helsly *et al.* 2022), although in those instances, dogs had only been exposed to stressful situations, not painful interventions.

Bitches in our study displayed behaviours associated with stress upon arrival at the clinic as they were separated from their owners and placed in isolation within the ICU. These actions have been shown to contribute to elevated stress levels in dogs in hospitalisation settings (Siracusa *et al.* 2008, 2010; Srithunyarat *et al.* 2016; Lloyd 2017; Edwards *et al.* 2019). However, surprisingly, upon arrival at the clinic, differences were detected in the percentage of bitches in the VPS and NVPS groups displaying the ‘comfortable’ and ‘restless’ CMPS-SF-associated descriptive items. The observed differences between the two groups at T1, coupled with our approach of allowing owners to decide whether to visit their dogs after ovariohysterectomy (OVH) upon arrival at the clinic, may suggest a relationship between owners who opted to see their dogs and the psychological stress experienced by the dogs when separated. Previous research has highlighted the mutual perception and emotional interplay between owners and dogs (Handlin *et al.* 2012; Buttner *et al.* 2015; Petersson *et al.* 2017; Sundman *et al.* 2019). It is plausible to hypothesise that owners who chose to visit their dogs might have stronger attachments to them and could experience heightened anxiety when leaving them at the clinic. This emotional bond could potentially contribute to the observed differences in the CMPS-SF-associated descriptive items between the groups.

While interpreting our findings, it is important to exercise caution due to the limited number of animals included and the potential lack of standardisation resulting from the study’s group selection approach based upon owners’ decisions. However, concurrently examining the impact of owners on their dogs’ POPPS and the inactivity rate using two distinct parameters, coupled with the statistical similarity observed in both, enhances the reliability of the study.

## Animal welfare implications and Conclusion

While challenges in spaying and neutering pain control have been extensively studied, achieving consistent and effective prevention remains elusive. The findings in this paper suggest that owner-assisted pain and stress management may prove beneficial across clinical settings. This study delves into the impact of positive dog-owner interaction on POPPS and inactivity rate in bitches undergoing ovariohysterectomy (OVH). The aim is to highlight the potential clinical advantages of such interactions in POPPS management. Our hypothesis, which posited that positive dog-owner interaction after surgery could significantly alleviate POPPS in OVH-operated bitches, found robust support in statistically significant results. The importance of owners’ presence and interaction with their dogs in mitigating POPPS, potentially by serving as emotional and cognitive distractors, was further reinforced by observing the same effect across highly effective and less effective analgesia/anaesthesia protocols.

Notably, alterations in the ‘Happy and Content or Happy and Bouncy’, ‘Quiet’, and ‘Nervous/Anxious/Fearful’ CMPS-SF-associated descriptive items emerged as the most prominent variables influenced by dog-owner interaction or its absence. These findings align with the understanding that these items carry a profound emotional component, underscoring the well-established affective bond between dogs and humans. Bitches that engaged in a 45-min interaction with their owners and experienced reduced POPPS also demonstrated a decrease in inactivity rate, which further underscored the role of this interaction in promoting a more comfortable and emotionally enriched post-operative recovery process for dogs. Exploring the role of owners in post-surgery pain relief presents an avenue for ground-breaking research. Investigating these interactions’ emotional, behavioural, and physiological aspects can deepen our comprehension of the human-dog bond and may pave the way for innovative post-operative pain management strategies.
